# Extensive Fracture of the Cranium, with Extravasation of Blood. Death Occurring in Fifteen Hours

**Published:** 1853-05

**Authors:** James M. Newman


ART. III. — *Extensive Fracture of the Cranium, with Extravasation of Blood, Death occurring in fifteen hours.* Read before the Buffalo Medical Association. By JAMES M. NEWMAN, M. D.

On Sunday, the 23d of January, 1852, about 8 o'clock, A. M., a man by the name of John Malony, was received into the Erie County Penitentiary, in a state of complete insensibility. He had been committed by a Watch-House Justice, for twenty-five days, on the charge of drunkenness, though he was completely insensible at the time of his sentence, and unable to give any account of himself. The constable accompanying him, pronounced him "dead drunk."

He had been conveyed nearly a mile in an open wagon, upon a chilly morning, and was carried into the Penitentiary apparently more dead than alive. He was cold and nearly pulseless; breathed feebly; face congested; speechless, and unconscious of every thing.

He was placed upon a bed near the fire, warmth applied to the extremities, frictions used, and stimulants administered. No benefit resulted from these applications, other than a return of warmth to the surface, an increase of the pulse, and of the strength of respiration.

I saw him about 2½ o'clock, P. M. I found him unconscious; pulse about 125 per minute; breathing stertorous; pupils dilated and insensible to light; eyelids drooped; the conjunctiva injected; the lips of a dark-blue color, but the face blanched and pale. I could obtain no other information as to the cause of this train of symptoms than that already given. His pulse evidently failed while I was examining him. I did nothing, and he died in about half an hour after I first saw him. There was not the least mark or sign upon the face or head, to indicate that he had received injury.

From a prisoner who had also been committed the same day, we learned that he had been placed in the same cell, in the watch-house, with Malony, about 12 o'clock the previous night, and that he found him then insensible, and lying upon the floor, and that he had vomited. He endeavored to arouse him, but without success, and he continued equally insensible during the night.

The wife of the dead man asserted that her husband was well the previous evening, but admitted that he had been drinking some during the day. His brother stated that he was at his house near the Niagara Falls car-house, until between nine and ten o'clock the previous evening, when he left for his own home, and he insisted that he was sober at the time.

A coroner's jury was summoned, when the following facts were elicited:

*Post-mortem examination;* about 10 o'clock Monday morning, some eighteen hours after death, assisted by Dr. Eastman. Attention was directed to the head. Externally it presented no mark of contusion, or injury, except that the right ear was deeply colored. About an inch above, and a little to the right of the right eye-brow, a point *seemed* more depressed than the parts surrounding, and suggested the possibility of this being the point of a fracture.

Upon dissecting off the scalp, the parts over the right temporal muscle, and the muscle itself, together with the parts over the occiput, were deeply infiltrated with blood.

Upon removal of the calvaria, a fracture was found commencing about three-quarters of an inch above, and a little to the right of the superciliary ridge, involving both plates of the frontal, temporal, parietal and occipital bones of that side, extending to the base of the cranium. The internal portion of the *os temporis* was driven in upon the brain.

A second fracture, through the inner plate only, existed in the parietal bone about an inch above the first fracture, extending in length about one half of the distance of the first.

Blood had been largely effused upon the brain, external to the dura mater, forming a large, firmly coagulated mass, weighing, as was estimated, some four ounces, and compressing the brain so firmly, as to have reduced the right hemisphere to nearly one half of its normal size. Numerous dark coagulated masses were to be seen beneath the dura mater, studding the entire surface of the brain, giving it a dark, mottled appearance.

The brain was not cut into, or removed, or the examination further prosecuted, as we had found sufficient cause of death, and the jury were in waiting, and the friends of the deceased were becoming clamorous for the removal of the body.

Before the coroner's jury, the testimony disclosed the facts that Malony was seen about 10 o'clock the evening previous to his death, intoxicated; that at near midnight he went to the front door of a dwelling-house on Franklin street, and rang the bell; and being asked, from an upper window, what he wanted, he looked up to reply, and in doing so fell over backward from the stoop, upon the edge of which he was standing, falling upon the pavement beneath, a distance of some four or five feet. Not moving from the position in which he fell, a watchman was summoned, and the man conveyed, in an insensible state, to the watch-house; and in the morning, without any attention being given to, or examination made of his case, he was convicted and punished for the crime of drunkenness.

The points of interest in the case are, the absence of any mark or injury; the extent of the fractures; and the amount of the effusion of blood.*

---

* We would add, as another point of interest in the case, the detention in a watch-house cell all night, no medical examination being requested, and the trial, conviction and sentence the following morning, of a person all this time in profound coma! In this aspect the case presents a striking commentary on the summary administration of justice! We trust, for the credit of our city, as well as for the sake of humanity, such truly horrible instances are of infrequent occurrence.—Editor Buffalo Med. Jour.

